# Down‐regulation of BnDA1, whose gene locus is associated with the seeds weight, improves the seeds weight and organ size in *Brassica napus*

**DOI:** 10.1111/pbi.12696

**Published:** 2017-02-20

**Authors:** Jie‐Li Wang, Min‐Qiang Tang, Sheng Chen, Xiang‐Feng Zheng, Hui‐Xian Mo, Sheng‐Jun Li, Zheng Wang, Ke‐Ming Zhu, Li‐Na Ding, Sheng‐Yi Liu, Yun‐Hai Li, Xiao‐Li Tan

**Affiliations:** ^1^ Institute of Life Sciences Jiangsu University Zhenjiang China; ^2^ The Oil Crops Research Institute (OCRI) of the Chinese Academy of Agricultural Sciences (CAAS) Wuhan China; ^3^ State Key Laboratory of Plant Cell and Chromosome Engineering Institute of Genetics and Developmental Biology (IGDB) Chinese Academy of Sciences (CAS) Beijing China

**Keywords:** Association analysis, *B. napus*, *DA1*, Overexpression, Seed size

## Abstract

*Brassica napus L*. is an important oil crop worldwide and is the main raw material for biofuel. Seed weight and seed size are the main contributors to seed yield. DA1 (DA means big in Chinese) is an ubiquitin receptor and negatively regulates seed size. Down‐regulation of AtDA1 in *Arabidopsis* leads to larger seeds and organs by increasing cell proliferation in integuments. In this study, BnDA1 was down‐regulated in *B. napus* by over expressed of *AtDA1* ^*R358K*^, which is a functional deficiency of DA1 with an arginine‐to‐lysine mutation at the 358th amino acid. The results showed that the biomass and size of the seeds, cotyledons, leaves, flowers and siliques of transgenic plants all increased significantly. In particular, the 1000 seed weight increased 21.23% and the seed yield per plant increased 13.22% in field condition. The transgenic plants had no negative traits related to yield. The candidate gene association analysis demonstrated that the *BnDA1* locus was contributed to the seeds weight. Therefore, our study showed that regulation of DA1 in *B. napus* can increase the seed yield and biomass, and DA1 is a promising target for crop improvement.

## Introduction

Rapeseed (*Brassica napus L*.) is an important oil crop. Its contribution to global oilseed production is considerable, and approximately 71.0 million metric tonnes were produced worldwide in 2014 (data from FAOSTAT http://faostat3.fao.org/browse/Q/QC/E). Although the yield of rapeseed is high, the supply of rapeseed oil is insufficient globally. Furthermore, market demand for vegetable oil‐derived biodiesel is increasing rapidly because the amount of available fossil fuels is decreasing dramatically (Sidibe *et al*., 2010). Therefore, there is a need to maximize the productivity of vegetable oil to solve the problem of edible oil supply and relieve pressure on energy supply (Chauhan *et al*., 2012). Rapeseed oil is a crucial source of vegetable oil, and currently, many studies have focused on enhancing the synthetic activity of oil to elevate the oil content (Li *et al*., 2013b, Tan *et al*., 2014). However, lipid synthesis efficiency is limited, and achieving oil content to 50%–55% in *B. napus* seems to be a natural limit (Li *et al*., 2006). Increasing the biomass of rapeseed could be an alternative way of increasing the total oil when oil content remains constant.

Seed weight is a main yield component and also a key trait that influences seedling establishment and seed dispersal (Gegas *et al*., 2010; Kesavan *et al*., 2013; Zhang *et al*., 2015). The seedlings of large‐seeded plants are capable to adapt the stressful environment, while small‐seeded plants are thought to produce large numbers of seeds (Moles *et al*., 2005; Westoby *et al*., 2002). The seed and organs size is regulated by both cell number and cell size, which are controlled by coordinating cell proliferation and cell expansion during organogenesis (Mizukami, 2002; Sugimoto‐Shirasu and Roberts, 2003). The mechanism that regulated the seed size and weight was well studied in *Arabidopsis*. For example, phytohormone signalling pathway is involved in the seed size regulation, and cytokinin acts downstream of the IKU pathway to regulate seed size (Li *et al*., 2013a), while *iku* mutations reduce seed size due to precocious cellularization of the endosperm (Garcia *et al*., 2003; Luo *et al*., 2005; Wang *et al*., 2012). AUXIN RESPONSE FACTOR2 (ARF2) regulates seed cell proliferation in the integuments to affect seed size (Schruff *et al*., 2006). Other factors also influence the seed size. KLUH/CYTOCHROME P450 78A5 (CYP78A5) affects seed size by promoting cell proliferation in the integuments (Adamski *et al*., 2009). In other side, TRANSPARENT TESTA GLABRA2 (TTG2) promotes cell elongation in the integuments to increase the seed size (Garcia *et al*., 2005). On the contrary, APETALA2 (AP2) represses cell elongation in the integuments to suppress the seed size (Jofuku *et al*., 2005; Ohto *et al*., 2005, 2009). Overexpression of CYP78A6 promotes seed size by both increasing the cell proliferation and cell elongation in the integuments (Fang *et al*., 2012). Seed size is also influenced by zygotic tissues. SHORT HYPOCOTYL UNDER BLUE1 (SHB1) promotes endosperm proliferation to increase seed growth (Zhou *et al*., 2009). In addition, the endosperm growth is also affected by epigenetic mechanisms (Xiao *et al*., 2006).

The ubiquitin receptor DA1 restricts cell proliferation in the integuments to affect seed size (Li *et al*., 2008; Xia *et al*., 2013). Mutations in EOD1, which encodes the E3 ubiquitin ligase (Disch *et al*., 2006; Li *et al*., 2008), synergistically promote the seed size of *da1‐1*, showing that DA1 acts synergistically with EOD1/BB to regulate seed size. On the whole, factors that regulate the seeds size have successfully characterized, and many works have been reported. However, there was only one paper published on seed size in *B. napus* (Liu *et al*., 2015).

The network for controlling seed size has been well described in model plants like *Arabidopsis*. But the similar study has not been reported in rapeseeds. AtDA1 negatively regulates seed and organ size, and the phenotype of the *da1‐1* mutant shows large seeds and organs in *A. thaliana* (Li *et al*., 2008). Then, we overexpressed deficient *AtDA1* (*AtDA1* ^*R358K*^) in rapeseed to investigate whether larger seed sizes and higher seed yields could be obtained and confirm the potential way to improve oil crop yields.

Candidate gene association analysis is based on polymorphism at the DNA level. It is used to discover alleles that make large contributions to the target traits from the natural population and is helpful to further validate gene function and dissect the site of the key role. Additionally, it can be used to analyse multiple effects for pleiotropic genes (Chen and Lubberstedt, 2010). This method was successfully applied to discover the *Dwarf8* (Thornsberry *et al*., 2001) and *Vgt1* (Salvi *et al*., 2007) polymorphisms associated with variation in flowering time. In addition, association analysis of flowering time genes *Hd1*,* Hd3a* and *Ehd1* showed that the Hd1 protein type, *Hd3a* promoter and *Ehd1* expression level were major factors in rice flowering (Takahashi *et al*., 2009). However, association analysis has not been used to validate gene function in *B. napus* to our knowledge. In this work, candidate gene association analysis was also used to verify the contribution of *BnDA1* to seed weight in a natural *B. napus* population.

## Results

### BnDA1 is highly homologous with AtDA1

**Figure 1:**
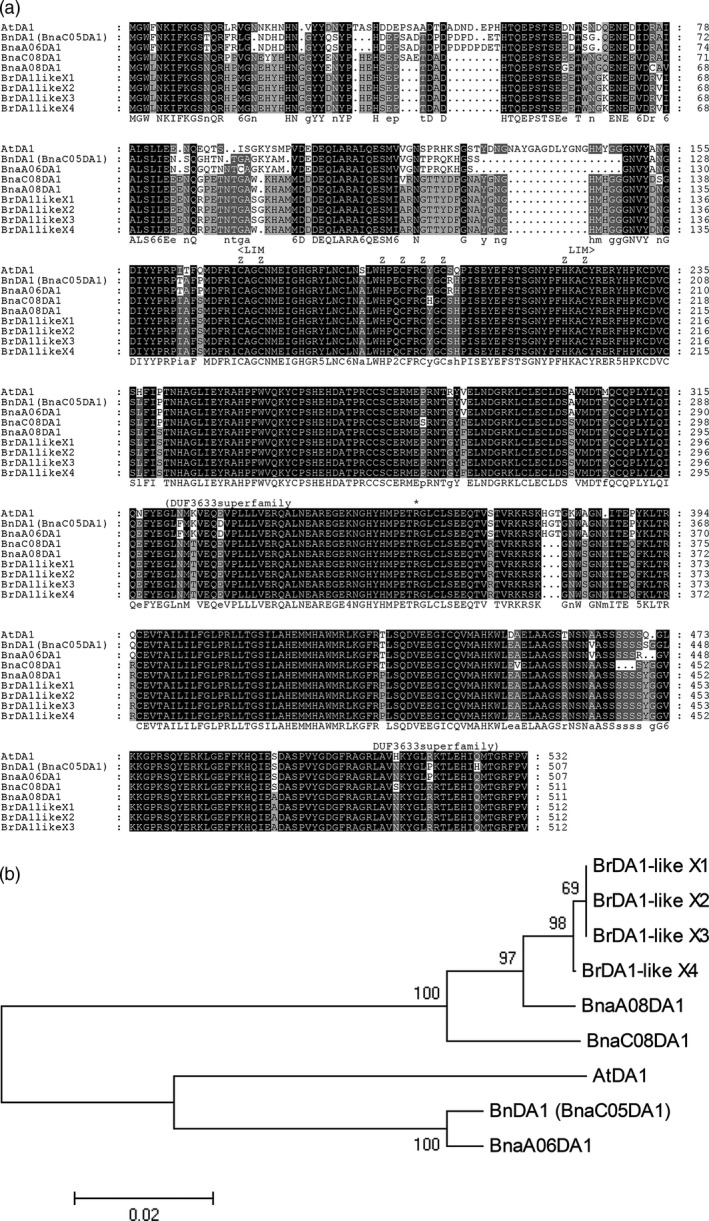
Analysis of the BnDA1 and AtDA1 amino acids sequences. (a) Multiple sequence alignments of the amino acid sequences. Proteins BrDA1‐like X1 to BrDA1‐like X4 come from *B. rapa*. They are DA1‐like isoform proteins. The proteins BnaC08g18690D, BnaA08g22120D and BnDA1 (BnaC05g14930D) come from *B. napus*. They are DA1 related or have similar functions. AtDA1 is from *A. thaliana*. (b) Phylogenetic tree for BnDA1, AtDA1 and DA1‐like protein from *B. rapa* and *B. napus*. The picture was constructed by the neighbour‐joining method using IMAGE5.2.

BnDA1 (BnaC05g14930D) and AtDA1 contain 507 and 532 amino acids, respectively; they share 83.15% identity (Figure S1). BnDA1 contains the LIM‐DA1 domain, corresponding to the ‘<LIM···LIM>‘ in Figure 1a, and Zn binding sites, which are located in the LIM‐DA1 domain. BnDA1 has another domain (DUF3633 superfamily), which corresponds to the ‘(DUF3633···DUF3633)’ in Figure 1a. This domain family is found in bacteria and eukaryotes. This functional domain is very conservative in BnDA1 and AtDA1. The mutation site for AtDA1^R358K^ is in the domain of the DUF3633 superfamily (The ‘*’ shown in Figure 1a). Showing that the DUF3633 functional domain is related to the activity of DA1.

The phylogenetic tree analysis, based on the amino acid sequences, showed that BnDA1 and AtDA1 were in one clade (Figure 1b). DA1‐like isoform proteins, BrDA1‐like X1, BrDA1‐like X2, BrDA1‐like X3 and BrDA1‐like X4, are selected from *B. rapa*. The proteins BnDA1 (BnaC05DA1), BnaAO6DA1, BnaC08DA1 and BnaA08DA1 are all from *B. napus*. BnDA1 has similar domains with AtDA1. BnDA1 and AtDA1 also have the highest similarity in amino acid levels (Figures 1a, S1), so we named it BnDA1. From the sequence analysis, we can show that BnDA1 and AtDA1 are closely related in terms of amino acid sequence and evolutionary relationship. This means that they are also likely to have similar functions.

### Overexpression of *BnDA1* can recover the *da1‐1* phenotype in *Arabidopsis thaliana*

To further prove that the function of BnDA1 and AtDA1 is conserved, 35S promoter::*AtDA1* and a 35S promoter::*BnDA1* expression vectors were constructed and transformed into the *Arabidopsis* mutant *da1‐1*. We found that the *da1‐1* phenotype was recovered in leaves (Figure S2a), flowers (Figure S2b) and seeds (Figure S2c). The complementary assays revealed that BnDA1 and AtDA1 had similar functions in regulating seed and organ size in *A. thaliana,* and potentially in *B. napus*.

### The analysis of expression level of *DA1* in *AtDA1* ^*R358K*^ overexpression lines in *B. napus*

**Figure 2:**
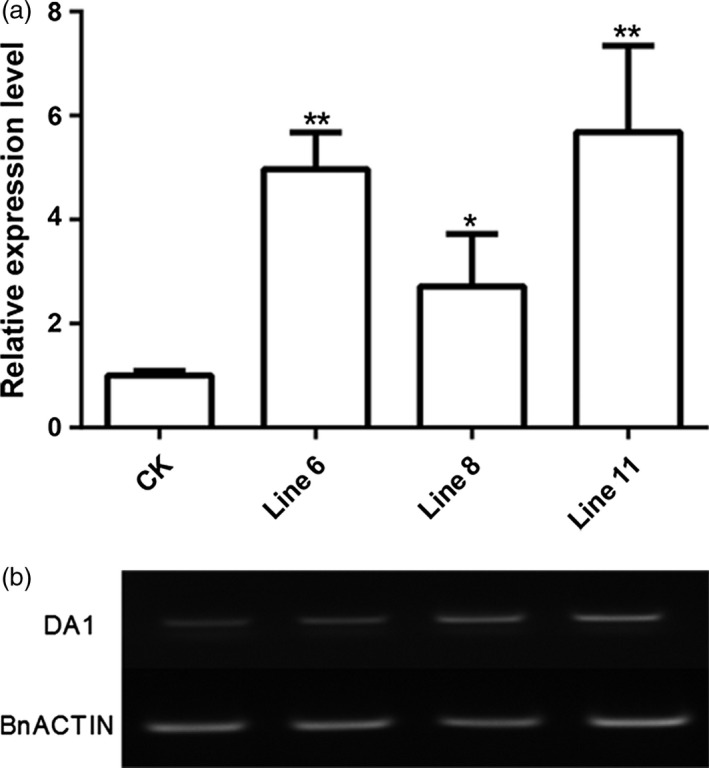
Relative expression levels of *AtDA1* in *AtDA1* ^*R358K*^ transgenic plants. (a) Relative expression levels of *AtDA1* in homozygous *AtDA1* ^*R358K*^ transgenic plants and CK were quantified by quantitative real‐time PCR. The quantity of each transcript was measured using the 2^−▵▵Ct^ method. *BnACTIN2* was used as an internal control. Value represents mean ±the standard error from three independent rapeseed samples. (b) Semi‐quantitative RT‐PCR also showed that *AtDA1* expression levels significantly increased in Line 6, Line 8 and Line 11 compared to CK, *BnACTIN2* was also used as an internal control. Value represents mean ± standard error from three independent rapeseed samples. * means a significant difference at the *P* < 0.05 level, and ** represents a significant difference at the *P* < 0.01 level.

As overexpression of *AtDA1* ^*R358K*^ resulted in large seed and organs (Li *et al*., 2008; Weng *et al*., 2008), we overexpressed the deficient *AtDA1* ^*R358K*^ in rapeseed to verify the function of AtDA1 in *B. napus* and to obtain potentially larger seeds. The binary vector containing *35S::AtDA1* ^*R358K*^ was transformed into wild‐type (WT) rapeseed plants by floral dipping approach (Li *et al*., 2010). The transgenic plants were identified by PCR (the primers were shown in Table S1 and the PCR product is shown in Figure S3). We further identified the expression levels of *AtDA1* by real‐time quantitative PCR (qRT‐PCR) and RT‐PCR. Among *AtDA1* over expression lines, Line 6 showed an almost threefold higher expression, and Line 8 and Line 11 were more than fivefold higher than in the WT (Figure 2a). Therefore, these three lines were chosen for further phenotype analysis. RT‐PCR analysis also produced identical results to qPCR (Figure 2b). Due to the high sequence similarity between *AtDA1* and *BnDA1*, the set of primers chosen could bind both the *AtDA1* and *BnDA1* sequences (Figure S4). Thus, the WT control (CK) had the background in qPCR and RT‐PCR analysis. Through expression analysis, we confirmed that the higher expression level of *AtDA1* ^*R358K*^ lines in *Brassica napus* was obtained.

### Overexpression of *AtDA1* ^*R358K*^ increase thousand seed weight (TSW) and organs size in rapeseed

**Figure 3:**
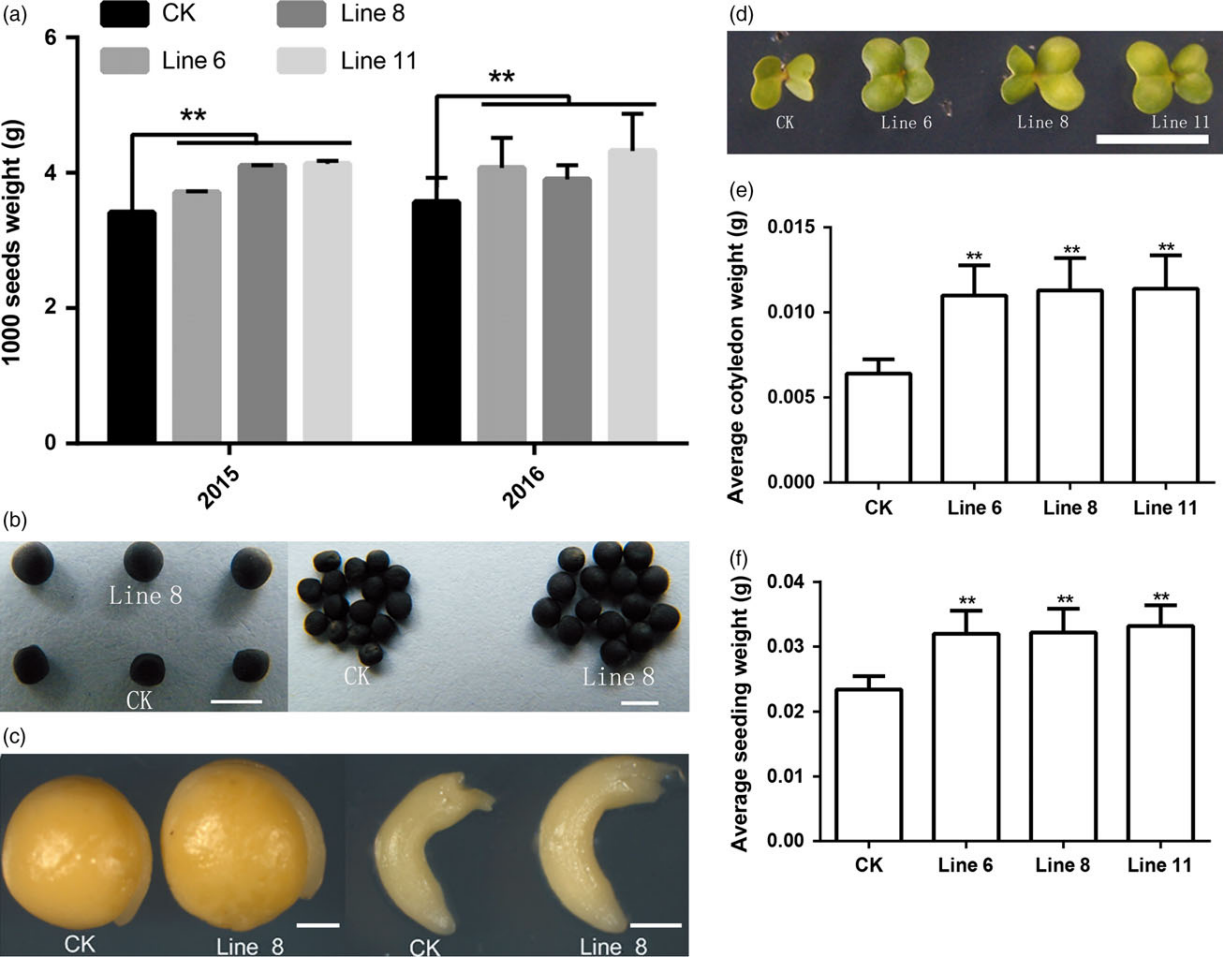
Overexpression of *AtDA1* ^*R358K*^ increases the weight and size of the seeds. (a) The 1000‐seed average weights of CK and the transgenic plant lines 6, 8 and 11. Standard deviations are shown (*n* = 5). (b) The seeds from transgenic plant line 8 were compared to seeds from CK. Bar = 2 mm. (c) A comparison of the embryo and hypocotyl from CK and transgenic plant line 8. (d) The 3‐d‐old seedlings of CK and the transgenic plant lines 6, 8 and 11, Bar = 5 mm. (e) The average cotyledon weight of CK and the transgenic plant lines 6, 8 and 11. (f) The average seedling weight and the seedling average weight. Value represents mean ± standard error from three independent rapeseed samples. ** represents significant differences at the *P* < 0.01 level.

The TWS of 2 years data (Figure 3a) showed that the seed size of the transgenic lines was significantly larger than the CK seed. The TWS of line 11 was 21.23% higher than CK (Figure 3a, b). The size of a seed is regulated by the coordinated growth of the embryo, endosperm and maternal tissue. We therefore examined the size of the embryo and hypocotyl and found that the sizes of the embryo and hypocotyl in transgenic lines had also increased compared to CK (Figure 3c). These results suggested that overexpression of *AtDA1* ^*R358K*^ increased the seed weight and size in *B. napus*.

**Figure 4:**
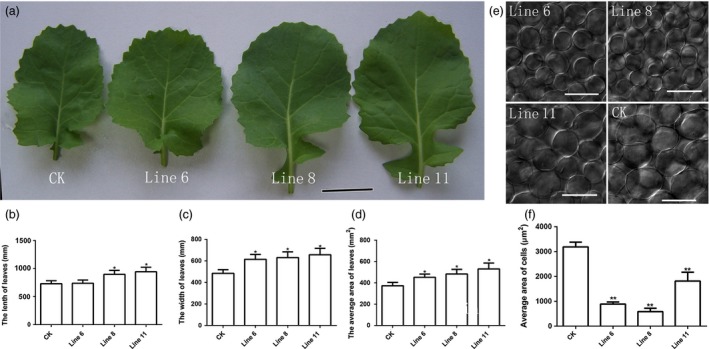
Overexpression of *AtDA1* ^*R358K*^ increases the size of leaf. (a) The leaves of CK and the transgenic plant lines 6, 8 and 11. Bar = 1 cm. (b) The leaf lengths of CK and transgenic plant lines 6, 8 and 11. (c) The leaf widths of CK and transgenic plant lines 6, 8 and 11. (d) The leaf average areas of CK and transgenic plant lines 6, 8 and 11. (e) The palisade cells of CK and the transgenic plant lines 6, 8 and 11, Bar = 50 μm. (f) The palisade cell average size of CK and the transgenic plant lines 6, 8 and 11. The leaves were collected from 24‐d‐old seedlings. Value represents mean ± standard error from three independent rapeseed samples.* means a significant difference at the *P* < 0.05 level, and ** represents a significant difference at the *P* < 0.01 level.

The seed size also affected the seedlings size. After the seeds germinated, the cotyledon weight and area of 3‐d‐old seedlings from the three selected transgenic lines were measured. The results showed that overexpression of *AtDA1* ^*R368K*^ increased cotyledon size compared to CK. Cotyledons of the 3‐d‐old transgenic line were significantly larger than the wild‐type cotyledons (Figure 3d and e). The biomass data for the seedlings showed that the seedling weight had also increased (Figure 3f). Therefore, the overexpression of *AtDA1* ^*R358K*^ increased the seed weight and size and further enhanced the size of the cotyledons. We also collected the biomass data for leaves, which showed that overexpression of *AtDA1* ^*R358K*^ produced large leaves compared to CK. The leaves of the transgenic lines were also more rounded than CK (Figure 4a), like those observed in *Arabidopsis da1‐1* mutant. We further measured the length, width and area of 24‐d‐old leaves. The results demonstrated that they had all increased (Figure 4b, c and d). The leaf palisade cell sizes of CK and *AtDA1* ^*R358K*^ overexpression plants were then measured to evaluate whether the leaf size increase in *AtDA1* ^*R358K*^ overexpression plants was due to increased cell proliferation. The results showed that the palisade cells in *AtDA1* ^*R358K*^ overexpression plants were significantly smaller than in CK (Figure 4e). The average area of palisade cells in *AtDA1* ^*R358K*^ overexpression plants was about 70% smaller than CK (Figure 4f). This implied that the larger leaf was caused by an increase in the number of cells in *AtDA1* ^*R358K*^ overexpression plants.

**Figure 5:**
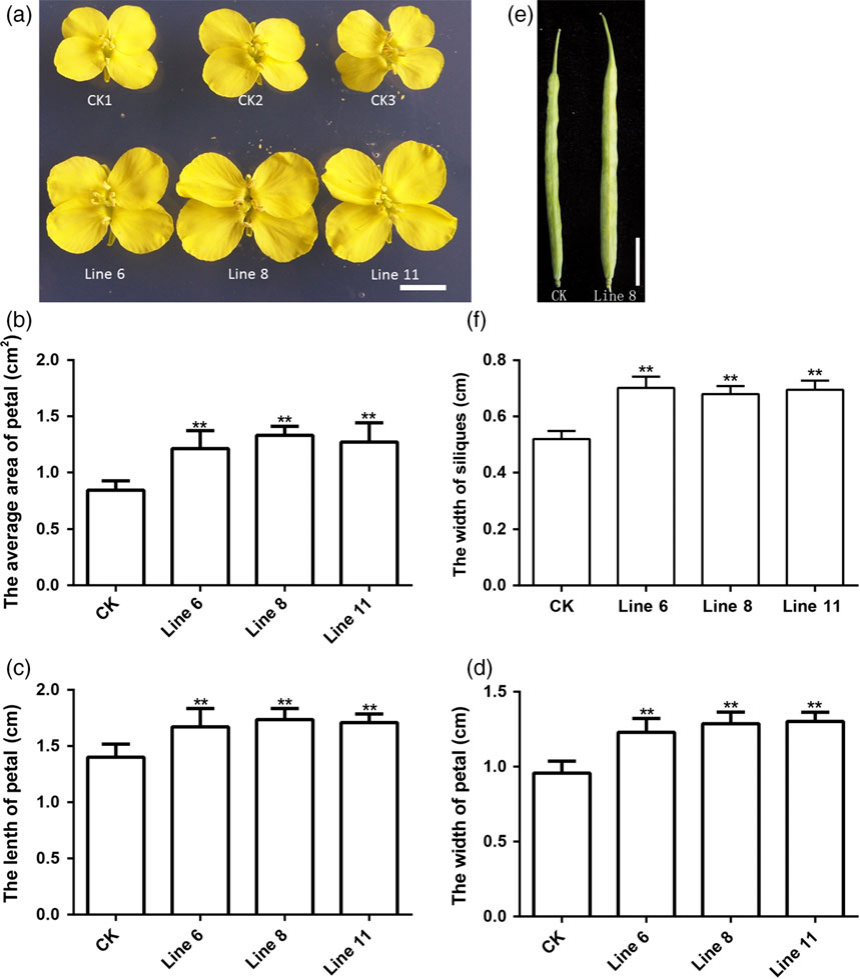
Overexpression of *AtDA1* ^*R358K*^ increases the size of the flowers and siliques. (a) The flowers of CK and the transgenic plant lines 6, 8 and 11. Bar = 1 cm. (b) The petal average areas of CK and the transgenic plant lines 6, 8 and 11. (c) The petal average lengths of CK and the transgenic plant lines 6, 8 and 11. (d) The petal average width of CK and the transgenic plant lines 6, 8 and 11. (e) The siliques of CK and the transgenic plant line 8. Bar = 2 cm. (f) The siliques average width of CK and the transgenic plant lines 6, 8 and 11. Standard deviations are shown (*n* = 10). Value represents mean ± standard error from three independent rapeseed samples. ** represents a significant difference at the *P* < 0.01 level.

The flower is one of the reproductive organs, and it is a major feature of higher plants. Flower pollination is an important process during sexual reproduction in flowering plants. As shown in Figure 5, the flowers were larger on the *AtDA1* ^*R358K*^ overexpression plants compared to CK (Figure 5a). The area, length and width of the petals increased significantly (Figure 5b, c and d), indicating that overexpression of *AtDA1* ^*R358K*^ had an important influence on flower development, but the timing, frequency and duration of flowering have no difference.

After flowering, the siliques developed. We compared the size of the siliques between transgenic plants and CK. As expected, plants overexpressing *AtDA1* ^*R358K*^ formed wider siliques compared to CK (Figure 5e). The silique width of transgenic lines was noticeably wider than the width of CK (Figure 5f), but the number of seeds in the siliques did not differ.

### Overexpression of *AtDA1* ^*R358K*^ increases the seed yield per plant

**Figure 6:**
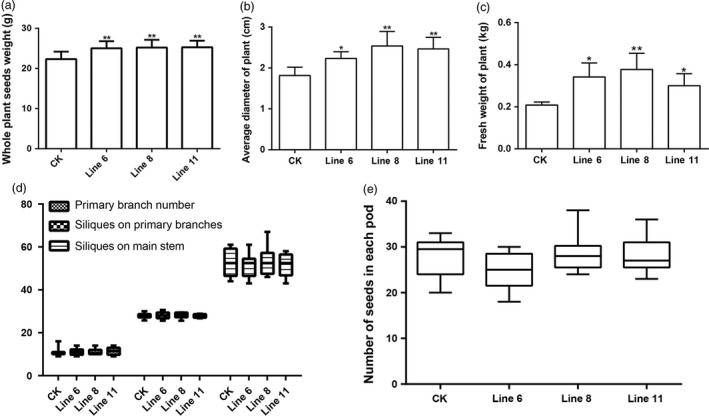
*AtDA1* ^*R358K*^ overexpression plants have no negative agronomic traits. (a) Whole plant seed weights of CK and transgenic plant lines 6, 8 and 11. (b) The single plant fresh weights of CK and transgenic plant lines 6, 8 and 11. (c) The plant average stem diameters of CK and transgenic plant lines 6, 8 and 11. The whole plant seed weights, the single plant fresh weight and the plant average stem diameter of transgenic plants significantly increased compared to CK. (d) Primary branches per plant, siliques on primary branches (siliques per primary branch) and siliques on main stem (Siliques per main stem) of CK and the transgenic plant lines 6, 8 and 11. (e) Number of seeds in each pod (Seeds per siliques) of CK and the transgenic plant lines 6, 8 and 11. Value represents mean ± standard error from three independent rapeseed samples. ** represents a significant difference at the *P* < 0.01 level.

The overexpression of *AtDAI* ^*R358K*^ leads to larger seeds, seedlings, embryos, cotyledons, leaves, flowers and siliques compared to CK. Apart from these positive agronomic traits, the transgenic plants also had other superior agronomic traits. The whole plant seed weights increased significantly. Line 11 increased by 13.22% compared to CK (Figure 6a), and the plant average stem diameter and plant fresh weight were also significantly higher (Figure 6b and c). This may lead to the observed increased yield and biomass of the transgenic plants compared to CK. Both the seed weights and biomass increased together (Figure 6a and c), and the final seed yield in the field could increase in a similar way. The over expression of *AtDA1* ^*R358k*^ increased the seed yield of a single plant, whether it accompanied by other negative agronomic traits. Therefore, we measured primary branch number per plant, silique number per primary branches, silique number per rachis and seed number per pod. These agronomic traits are the major components of rapeseed yield. The data showed that all these agronomic traits did not significantly change (Figure 6d and e). Therefore, overexpression of *AtDA1* ^*R358K*^ improved the seed yield of a single plant in filed without producing any negative agronomic traits.

### *BnDA1* homologous gene expression analysis and association analysis

**Figure 7:**
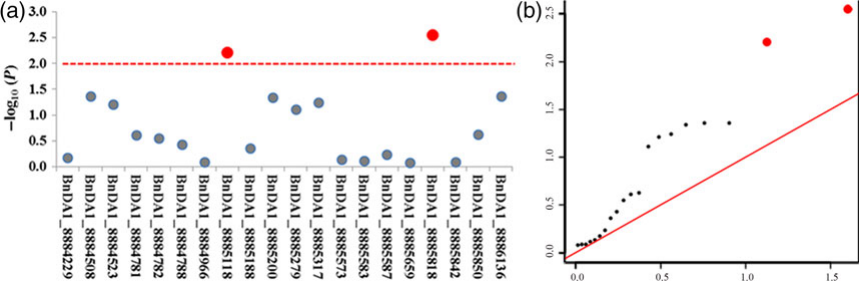
Association of SNP polymorphisms with TWS across the *BnDA1*. (a) The red‐dotted line was the significant threshold −log_10_(p) = −log_10_(0.01) = 2. 0; the red dot above the red‐dotted line represents a significantly associated SNP. (b) The Q‐Q plots for thousand seed weight (TSW) from association analysis. The red line was the unbiased estimates of the expected and observed value. Red dots were the significant SNPs associated with TSW based on threshold.

As *B. napus* is a recent allotetraploid species derived from *B. rapa* and *B. oleracea* (Naganara, 1935), it has many highly homologous genes. To identify BnDA1 as a major functional gene, transcriptome analysis was carried out on the unfolded petal and ovule. Compared to expression level of three other homologous genes mentioned in Figure 1b, the FPKM (Reads Per Kilobase of exon model per Million mapped reads) of BnDA1 was significantly higher and more than twice as high in both tissues (Figure S5), this indicated that BnDA1 gene was a major functional gene in the two tissues. Furthermore, to validate the function of *BnDA1* for TSW in other *B. napus* accessions, we performed association analysis of a set of 224 accessions collected from different geographic position, TSW varied from 2.83 to 5.52 g with an average of 3.88 g, and 20 polymorphism SNPs were detected in BnDA1 by re‐sequencing. The results displayed two significantly associated SNPs, BnDA1_8885118 and BnDA1_8885818, explaining 4.50% and 5.18% of TSW variation in this population, respectively (Figure 7a). For BnDA1_8885818, the corresponding line had the low TSW of 2.83 g, the SNP was in coding sequence of *BnDA1* and caused the wild‐type form of BnDA1, which negatively regulated the seed size. Finally, led to the lower TSW. In the QQ plot, the observed value of the two SNPs significantly deviates from expected value (Figure 7b), indicating that they were associated with TSW. Therefore, BnDA1 was the main functional gene among it homologues in the unfolded petal and ovule, and the BnDA1 locus in natural population was also associated with the seeds TSW.

## Discussion

Both *B. napus* and *A. thaliana* belong to the cruciferae family. The sequence of the functional genes is highly conserved (Jiang *et al*., 2015; Navabi *et al*., 2013). From the sequence analysis, we found that BnDA1 had a high sequence similarity with AtDA1 and that they have the same functional domain, suggesting that the BnDA1 and AtDA1 have conserved functions. The BnDA1 could functionally complement the *da1‐1* phenotype, providing further evidence that BnDA1 and AtDA1 have the same function with regard to regulating seed and organs size. Being able to produce larger seeds and organs in rapeseed oil crops has enormous economic value. If DA1 negatively regulates the seed size, then overexpression of the function deficient *AtDA1R* ^*358K*^ gene in rapeseed could compete with BnDa1, resulting in the down‐regulation of BnDa1 and the production of larger seeds and organs. Similarly, the protein encoded by *da1‐1* (*AtDA1* ^*R358K*^) has negative effects on DA1 and DA1‐related proteins (Kesavan *et al*., 2013; Wang *et al*., 2012; Zhao *et al*., 2015). Our experiments demonstrated that overexpression of AtDA1^R358K^ in rapeseed comprehensively enhanced the seed, cotyledon, leaf, flower and silique size. These results showed that BnDA1 and AtDA1 are functionally conserved. In addition, the larger seed and organ sizes have potential economic value in oil crop improvement and bioenergy production.

Overexpression of *AtDA1* ^*R358K*^ in rapeseed negatively regulated BnDA1, which led to larger seed and organs sizes. In agriculture, seed size is a main components of seed yield. Overexpression of *AtDA1* ^*R358K*^ in *B. napus* produced a 21.23% TSW increase and 13.22% increase in seed yield per plant. In addition, larger flowers help attract insects and improve pollination. Higher pollination efficiency could improve seed setting rate, which can directly affect the yield of flowering plants. In short, overexpression of *AtDA1* ^*R358K*^ in *B. napus* could increase rapeseed yield. In addition, the vegetative organs, such as seedlings, leaves and fresh weight of the whole plant, also increased in size without any negative influences on major agronomic traits and oil content.

The increased biomass production suggested that the DA1 gene has a potential application in the bioenergy industry and in cash crop improvement. However, we used a transgenic approach to achieve this goal, and the public have concerns about genetically modified organisms. The newly developed genome edit approach, CRISPR/Cas9, could circumvent public apprehension (Shan *et al*., 2014; Xu *et al*., 2015). Therefore, DA1 is a promising target for editing the genome with CRISPR/Cas9 because one amino acid alteration may produce the desired positive trait.

With the constant evolution of polyploid species, the expression and biological functions of homologous genes are possibly subject to subfunctionalization, and even produce neofunctionalization (Liu and Adams, 2007). Different homologous genes could be expressed in different tissues and organs at specific time. The homologous genes *TaWLHS1* (Shitsukawa *et al*., 2007) and *TaMBD2* (Hu *et al*., 2011) have different expression pattern in different tissues in wheat. In the transcriptome analysis, compared with three other homologous genes, *BnDA1* was shown as the major functional gene for unfolded petal and ovule, but whether it is also the major functional gene for other tissues requires further and analysis. Although the association analysis showed *BnDA1* was associated with TWS, the phenotypic contribution of the two significant SNPs detected by association analysis was lower than linkage mapping research (Fan *et al*., 2010). The reason may be that TSW is a complex quantitative trait controlled by many QTLs (genes) and that phenotypic variation is not wide enough (range from 2.83 to 5.52 g) in this natural population.

In conclusion, we have demonstrated that the overexpression of AtDA1^R358K^ with a single amino change in ubiquitin receptor DA1 caused larger seeds and organs in *B. napus*. The average TSW increased almost 21.23%, and seed production per plant increased by 13.22%. Other organs, including cotyledons, leaves, flowers and siliques, were also larger. These phenotype changes suggested that BnDA1 is a promising target for crop improvement and that it is feasible to edit BnDA1 with a CRISPR/Cas9 approach. Because the BnDA1 locus contributed to TSW of *B. napus* in the natural population, this locus could be developed as a functional molecular marker in marker assistant breeding for TSW improvement.

## Experimental procedures

### Plant materials and growth conditions

Rapeseed (*Brassica napus L*) *cv* Zhongshuang 9 (ZS9) was as the wild‐type control (CK). The ZS9 plants were planted in the experimental fields at Jiangsu University, Zhenjiang, China. *A. thaliana Landsberg erecta* (*Ler*) was also used as a WT. The plants were grown under long‐day conditions, which were 14‐h light at 22 °C and 10‐h dark at 20 °C.

### Sequence analysis of *BnDA1*

The *Arabidopsis thaliana DA1* gene was used to do a blast search of the *BnDa1* sequences and other closed genes in the NCBI. We identified a highly similar sequence in *Brassica napus L* named *Bnac05g14930D* (GenBank NO.), which we named BnDA1. Multiple sequence alignments of the amino acids sequences for AtDA1, BnDA1 and homologous proteins were performed using GeneDoc software. Phylogeny tests were accomplished using the bootstrap method with 1000 replications to reconstruct a neighbour‐joining tree using MEGA5.2 software. Pairwise deletion of gaps/missing data was employed, and uniform rates among sites and similar patterns among lineages were selected for the neighbour‐joining (NJ) trees.

### Vector constructs and plant transformation

The 35S::*AtDA1* and 35S::*BnDA1* constructs were made using a PCR‐based Gateway system (the PCR primers were shown in Table S1), according to the manufacture guide (Invitrogen, Carlsbad, CA). The *AtDA1* and *BnDA1* genes were subcloned into the Gateway binary vector pMDC32. The 35S::*AtDA1* and 35S:*BnDA1* plasmids were transformed with *Agrobacterium* GV3101, and the transformants were selected on the hygromycin‐contained medium. The pMDC32‐35S::*AtDA1* ^*R358K*^ vector was introduced into ZS9 according our previous report (Li *et al*., 2010).

### RNA isolation, RT‐PCR and q PCR analysis

Total RNA was extracted using TRIzol (Invitrogen, Carlsbad, CA), and mRNA was reverse transcribed using Revert Aid first‐strand cDNA Synthesis Kit (#K1622; Thermo Fisher Scientific, Dreieich, Germany). The samples of cDNA were standardized based on the amount of *BnACTIN* transcript using the primers *BnACTIN*‐F and *BnACTIN*‐R (Table S1). This pair of primers was also used in the RT‐PCR and q PCR analyses as a reference gene. The q PCR analysis was performed with SYBR Green format and SYBR premix Ex Taq II (Takara Biotechnology, Dalian, China) using the Applied Biosystems 7300 Fast Real‐Time PCR System (ABI, Carlsbad, CA). The primers used for RT‐PCR and q PCR were are described in Table S1‬‬‬‬‬‬‬‬‬‬‬‬‬‬

### Morphological analysis

Average seed weight was weighted by an electronic analytical balance (BS223S; Sartorius, Gottingen, Germany) with mature dry seeds in batches of 1000. The seeds were photographed using a camera (COOLPIXP7000; Nikon, Tokyo, Japan), and then, seed size, petals area and leaf area were measured using Image J software. Mature plant biomass accumulation was measured by weighing the different organs. The seedlings for analysis were planted in an incubator. The samples for analysis of fresh weight, plant average stem diameter, siliques on each branch, seeds number in each silique and seed oil content were all collected from the mature plants.

### Leaf and cell observations

Chloral hydrate was used to make the leaves transparent. Firstly, entire or small pieces of leaves were fixed in the same amount of absolute alcohol and glacial acetic acid mixture for 24 h. Secondly, the leaves were dipped in a saturated aqueous solution of chloral hydrate. Then, the leaves were washed carefully with pure water after the leaves were transparent. Finally, the leaves were floated on glycerol before observation. A metallurgical microscope (Axio Imager A1; ZEISS, Jena, Germany) was used to observe and record the cell size, and the data were analysed using Image J software.

### Analysis of seed oil content

Oil content was analysed by NMR (PC120; BRUKER, Karlsruhe, Germany). The data were classified and analysed via one‐way analysis of variance using the SAS statistical package (SAS Institute, Cary, NC). Comparisons between the treatment means were made using Duncan's multiple range test at the *P *< 0.05 level. Each line was measured three times.

### Association analysis of *BnDA1*

A worldwide collection of 224 rapeseed accessions was used for targeted gene association analysis. The phenotypic data were collected from the field experiments over 3 years in two locations (Wuhan, Hubei province and Yangzhou, Jiangsu province). Field experiments were designed in a randomized complete block design with three replicates and plot size of 3 m^2^. The open‐pollinated seeds were harvested from 10 individual plants of each plot when they were mature and measured for TSW. An R script (www.eXtension.org/pages/61006) based on a linear model was used to obtain the best linear unbiased prediction of TSW as phenotypic values in each line.

Genomic DNA was extracted from juvenile leaves of 224 self‐pollinated lines using the modified CTAB method. The polymorphic SNPs of *BnDA1* were genotyped by genomic DNA re‐sequencing. Association analysis was performed using the software Tassel 5.0.

### *BnDA1* homologous gene expression analysis

The total RNA from unfolded petal and ovule of cv. Zhongshuang 11 were extracted and then sequenced. The sequenced reads were mapped to the reference genome, and expression quantity was calculated as FPKM (Trapnell *et al*., 2010).

## Supporting information


**Figure S1** Sequence alignment of amino acids between AtDA1 and BnDA1. The first line is AtDA1, the second line is BnDA1, as drawn by DNAMAN 8. The similarity between the AtDA1 and BnDA1 sequences was 83.15%.

**Figure S2** Overexpression of *BnDA1* can recover the *da1‐1* phenotype. From left to right, the leaves, flowers, and petals are from *Col‐0*,* da1‐1*,* 35S::AtDA1*,* 35S::BnDA1‐1* and *35S::BnDA1‐9*, respectively. *35S::AtDA1*, and *35S::BnDA1* are all in the *da1‐1* background. (a) The fifth rosette grew out about 35 days after germination. (b) The fifth or sixth flower in bloom. (c) The petal of the fifth or sixth flower in bloom.

**Figure S3** The identification of *AtDA1* in *AtDA1* ^*R358K*^ transgenic plants by PCR. The primers used were 35S‐F2 in the 35S promotor and DA1‐R2 in the DA1 gene (Table S1). We picked out 11 independent transgenic lines (line4 to line6, line8 to line 11, and line13 to line16). The WT represents the CK (wild type of rapeseed), and the ‘‐’ is the negative control without any genome.

**Figure S4** Primer DA1‐QRTF (Above) and primer DA1‐QRTR (Below) for *AtDA1*. DA1‐QRTF and DA1‐QRTR were designed according to the nucleotide sequence. Both of them were 21 bp long and they can match the sequence of *AtDa1* and *BnDA1* sequence exactly.

**Figure S5** The expression level of *BnDA1* and three homologous genes in unfolded petals and ovule in Zhongshuang11 based on transcriptome analysis.

**Table S1** Quantitative real‐time RT‐PCR and identified PCR primers.
