# Supplementary figures and images for: Down‐regulation of BnDA1, whose gene locus is associated with the seeds weight, improves the seeds weight and organ size in Brassica napus
Source: Plant Biotechnol J. 2017 Feb 20;15(8):1024–33. doi: 10.1111/pbi.12696 (PMC5506660; doi:10.1111/pbi.12696)

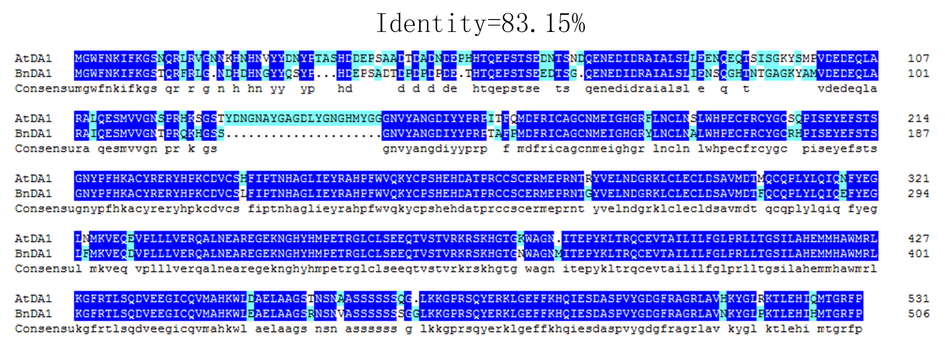

Supplement: Supplementary file 1 — Figure S1 Sequence alignment of amino acids between AtDA1 and BnDA1. The first line is AtDA1, the second line is BnDA1, as drawn by DNAMAN 8. The similarity between the AtDA1 and BnDA1 sequences was 83.15%. [file PBI-15-1024-s005.tif]

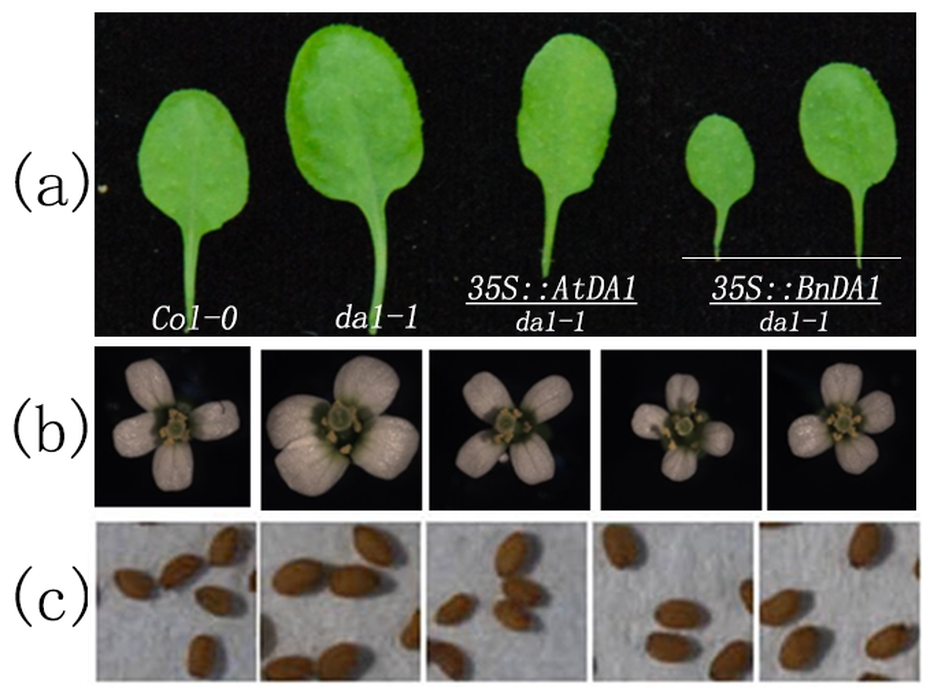

Supplement: Supplementary file 2 — Figure S2 Overexpression of BnDA1 can recover the da1‐1 phenotype. From left to right, the leaves, flowers, and petals are from Col‐0, da1‐1, 35S::AtDA1, 35S::BnDA1‐1 and 35S::BnDA1‐9, respectively. 35S::AtDA1, and 35S::BnDA1 are all in the da1‐1 background. (a) The fifth rosette grew out about 35 days after germination. (b) The fifth or sixth flower in bloom. (c) The petal of the fifth or sixth flower in bloom. [file PBI-15-1024-s004.tif]

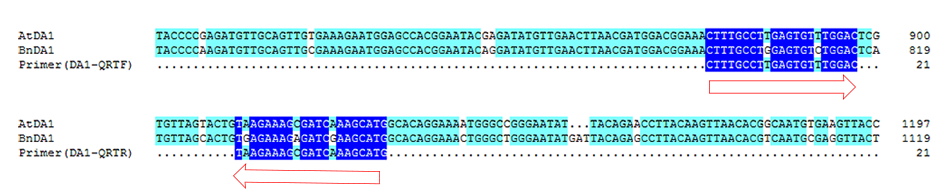

Supplement: Supplementary file 4 — Figure S4 Primer DA1‐QRTF (Above) and primer DA1‐QRTR (Below) for AtDA1. DA1‐QRTF and DA1‐QRTR were designed according to the nucleotide sequence. Both of them were 21 bp long and they can match the sequence of AtDa1 and BnDA1 sequence exactly. [file PBI-15-1024-s003.tif]

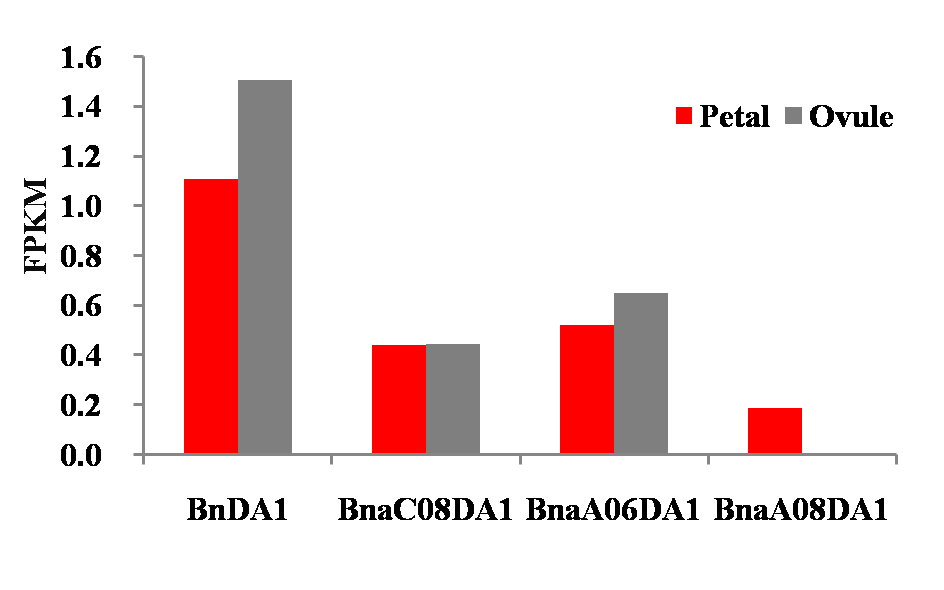

Supplement: Supplementary file 5 — Figure S5 The expression level of BnDA1 and three homologous genes in unfolded petals and ovule in Zhongshuang11 based on transcriptome analysis. [file PBI-15-1024-s002.tif]
