# Supplementary material for: Down‐regulation of BnDA1, whose gene locus is associated with the seeds weight, improves the seeds weight and organ size in Brassica napus
Source: Plant Biotechnol J. 2017 Feb 20;15(8):1024–33. doi: 10.1111/pbi.12696 (PMC5506660; doi:10.1111/pbi.12696)
Supplement: Supplementary file 6 — Table S1 Quantitative real‐time RT‐PCR and identified PCR primers. [file PBI-15-1024-s001.docx]

**Supplementary Table**

**Table S1.** Quantitative real-time RT-PCR and identified PCR primers

| Primer name | Sequence |
| --- | --- |
| AtDA1CDS-F | 5' ATGGGTTGGTTTAACAAGATCTT 3' |
| AtDA1CDS-R  BnDA1CDS-F  BnDA1CDS-R  DA1-QRTF | 5' TTAAACCGGGAATCTACCGGTC 3'  5' ATGGGTTGGTTTAACAAGATCTT 3'  5' TTAAACCGGGAATCTACCGGTC 3'  5' CTTTGCCTTGAGTGTTTGGAC 3' |
| DA1-QRTR | 5' CATGCTTTGATCGCTTTCTTA 3' |
| BnACTIN-F  BnACTIN-R  35S-F2  DA1-R2 | 5' GTTGCTATCCAGGCTGTTCT 3'  5' ACTGCTCTTAGCCGTCTCC 3'  5' CTTCGCAAGACCCTTCCTC 3'  5' AACAAAGAAAACACTGAATCT 3' |
